# Morphologic leukemia-free state in acute myeloid leukemia is sufficient for successful allogeneic hematopoietic stem cell transplant

**DOI:** 10.1038/s41408-021-00481-9

**Published:** 2021-05-16

**Authors:** Cindy M. Pabon, Zhiguo Li, Therese Hennig, Carlos de Castro, Jadee L. Neff, Mitchell E. Horwitz, Thomas W. LeBlanc, Gwynn D. Long, Richard D. Lopez, Anthony D. Sung, Nelson Chao, Cristina Gasparetto, Stefanie Sarantopoulos, Donna B. Adams, Harry Erba, David A. Rizzieri

**Affiliations:** 1 Division of Hematologic Malignancies and Cellular Therapy, Department of Medicine, Duke University Medical Center, Durham, NC USA; 2 Department of Biostatistics and Bioinfomatics, Duke University, Durham, NC USA; 3 Department of Pathology, Divisions of Hematopathology and Molecular Pathology, Genetics and Genomics, Durham, NC USA

**Keywords:** Cancer stem cells, Cancer stem cells

Dear Editor,

Survival for adults with acute myeloid leukemia (AML) has improved, however, relapse is common and it is standard to pursue allogeneic transplant (HCT) in such circumstances once the patient is in complete remission (CR)^1,2^. This presents a challenge for the clinician in that prior research has focused on transplantation while in remission, however, for those who may have an anti-leukemic response to therapy but not have full, durable count recovery, this definition does not apply. Furthermore, delays between chemotherapy and transplantation augment the chance of potentially life-threatening sequelae (i.e., infection and bleeding) and can allow for recrudescence of disease. Several studies have tried to address the question as to whether a formal “CR” for transplantation is necessary and have produced mixed results^3,4^. In addition, data on transplant outcomes presented from the Center for International Blood and Marrow Research (CIBMTR) database has been challenging to compare with non-transplant based studies as definitions of pre-transplant response differ from that of other widely utilized leukemia guidelines, such as the NCCN and Cheson criterion (Table 1)^5–7^. While definitions for CR are the same, CR with incomplete hematologic recovery (CRi) differs, and the CIBMTR does not recognize the morphologic leukemia-free state (MLFS). This difference in definitions results in misidentification of patients with MLFS as either having primary induction failure or persistence of relapsed disease. Further, some identified as CRi by CIBMTR, may in fact be MLFS and not CRi by NCCN criterion. Third, the MLFS definition also varies across groups, as is noted when comparing the original IWG 2003 Criteria to that of the current NCCN guidelines. While both require <5% blasts, the NCCN definition technically includes patients with anaplastic marrow^5^ without any signs of recovery. Thus, there are limited data on the efficacy of allogeneic HCT for patients who start allogeneic HCT in MLFS. Understanding post-transplant outcomes in this population are important to the clinician in counseling the patient as to the benefit of allogeneic HCT in this setting and in the timing for HCT. Therefore, we performed a retrospective review to elucidate the outcomes of patients with MLFS undergoing allogeneic HCT. We hypothesize that AML in MLFS is sufficient to justify allogeneic HCT and can result in long-term survival, thus obviating the need to delay a transplant until a formal complete remission is documented.

**Table 1:** Comparison of definitions for response criterion in AML from CIBMTR vs. NCCN vs. IWG2003.

CIBMTR		NCCN		IWG2003
**Complete remission** (must meet all criteria for at least 4 weeks): • <5% blasts in the bone marrow • No blasts with Auer rods • Normal maturation of all cellular components in the bone marrow • No extramedullary disease • Neutrophils ≥ 1000/µL • Platelets ≥ 100,000/µL • Transfusion independent	vs.	**Morphologic complete response** • Patient is independent of transfusions • Absolute neutrophil count > 1000/mcL (blasts < 5%) • Platelets ≥ 100,000/mcL (blasts < 5%) • No residual evidence of extramedullary disease **Cytogenetic complete response** Cytogenetics normal (in those with previously abnormal cytogenetics) **Molecular complete response** Molecular studies negative (only relevant in APL and Ph+ leukemia)	vs.	Morphologic complete response* • Patient achieves MLFS AND has ANC > 1000/µL AND platelets ≥100,000/µL • Transfusion independent Cytogenetic complete response* Cytogenetics normal (in those with previously abnormal cytogenetics) Molecular complete response* Molecular studies negative * For the three CR categories, extramedullary leukemia must be absent
**Complete remission with incomplete hematologic recovery (CRi)** • (must meet all criteria for at least 4 weeks): • Not meeting criterion for CR • <5% blasts in the bone marrow • No blasts with Auer rods • Normal maturation of all cellular components in the bone marrow • No extramedullary disease (e.g., CNS and soft tissue disease) • Transfusion independent (Please note, if the physician documents transfusion dependence related to treatment and not the patient’s underlying AML, CRi can be reported)	vs.	**Complete remission with incomplete hematologic recovery (CRi)** • This has been defined as <5% marrow blasts, and transfusion independence, but persistent cytopenia in the form of • ANC < 1000/mcL, OR • Platelets <100,000/mcL	vs.	**Morphologic complete remission with incomplete blood count recovery (CRi)** • Patients fulfill all criteria for CR except for residual neutropenia (<1000/µL) or thrombocytopenia (<100,000/µL)
**Primary induction failure (PIF)** • The patient received treatment for AML but never achieved CR or CRi at any time. • PIF is not limited by the number of unsuccessful treatments	vs.	**Induction failure** Failure to attain CR following exposure to at least 2 courses of intensive induction therapy		**Partial remission** Only relevant for phase I and II trials; all hematologic values for CR met but with a decrease of at least 50% in the blasts **Treatment failure** Patients who failed to achieve CR on phase III trial or less than PR on phase I or II trial
**Relapse** (After having attained a CR or CRi) (must meet one or more of the following): • ≥5% blasts in the marrow or peripheral blood • Extramedullary disease • Disease presence determined by a physician upon clinical assessment	vs.	**Relapse** Reappearance of leukemic blasts in the peripheral blood or the finding of more than 5% blasts in the bone marrow, not attributable to another cause.		**Recurrence** Reappearance of leukemic blasts in the peripheral blood or ≥5% blasts in the bone marrow
		**Morphologic leukemia-free state (MLFS)** • Bone marrow <5% blasts in an aspirate with spicules • No blasts with Auer rods or persistence of extramedullary disease • Not meeting criterion for CR or CRi	vs.	**Morphologic leukemia-free state** • Bone marrow <5% blasts in an aspirate with spicules PLUS > 200 nucleated cells • No blasts with Auer rods or persistence of extramedullary disease

## Methods

Through a standardized chart review of the 427 patients with AML who underwent allogeneic HCT at our center between 2005 and 2018, we identified those who fit the definition of MLFS, narrowing the population to 35. MLFS was defined using the published NCCN criterion, meeting all morphologic CR criteria except for absolute neutrophil count <1000/µL or platelet count <100,000/µL, but not both^5^. The study was designed by the authors and approved by the Duke University IRB.

Information reviewed included patient demographics, disease characteristics including risk strata per NCCN criteria (version 3.2020), donor and transplant details, relapse, and survival (CP and DR). Data were right-censored such that patients were assumed to be alive at the last time of follow-up, with the transplant team confirmation through May 2020 and assigning the cause of death. The cumulative incidence method was used to estimate relapse and NRM. Survival was illustrated through Kaplan–Meier curves.

## Results

We identified 35 adults meeting the criterion for MLFS, with a median age of 50 years (range: 20–72) with 29% meeting NCCN criteria for high-risk AML per NCCN guidelines^5,6^. The hematopoietic cell transplantation-specific comorbidity index (HCT-CI)^8^ median score was 4 (range: 1–9) (Supplementary Table 1). All patients were treated with aggressive induction attempts including 7 + 3, FLAG, or CPX-351. Despite this, all patients in the cohort had evidence of relapse or failure to attain CR after a minimum of two induction regimens. Nearly half (46%) of patients with cytogenetic data had no chromosomal abnormalities. Next-generation sequencing (NGS) data were only available for 17 patients. Seven had FLT3 mutations (ITD/TKD unknown), 4 with NPM1 mutations, and 6 with no abnormalities. Pre-transplant bone marrow cellularity was predominantly hypocellular at <5%. While all patients met MLFS by NCCN definition, the classification based on CIBMTR status was 54% PIF, 34% CRi, and 11% relapsed. As noted previously, the CIBMTR does not recognize MLFS and thus patients in this group are generally classified as relapsed, PIF, or CRi in that database.

Transplant characteristics may be reviewed in Supplementary data. Briefly, the median time from bone marrow biopsy/labs revealing aplastic status to transplant was 29 days, the conditioning regimens were reduced intensity in 11 of the 17, 25 donors were unrelated (7 cord blood), and 7 were haploidentical family members.

**Fig. 1:**
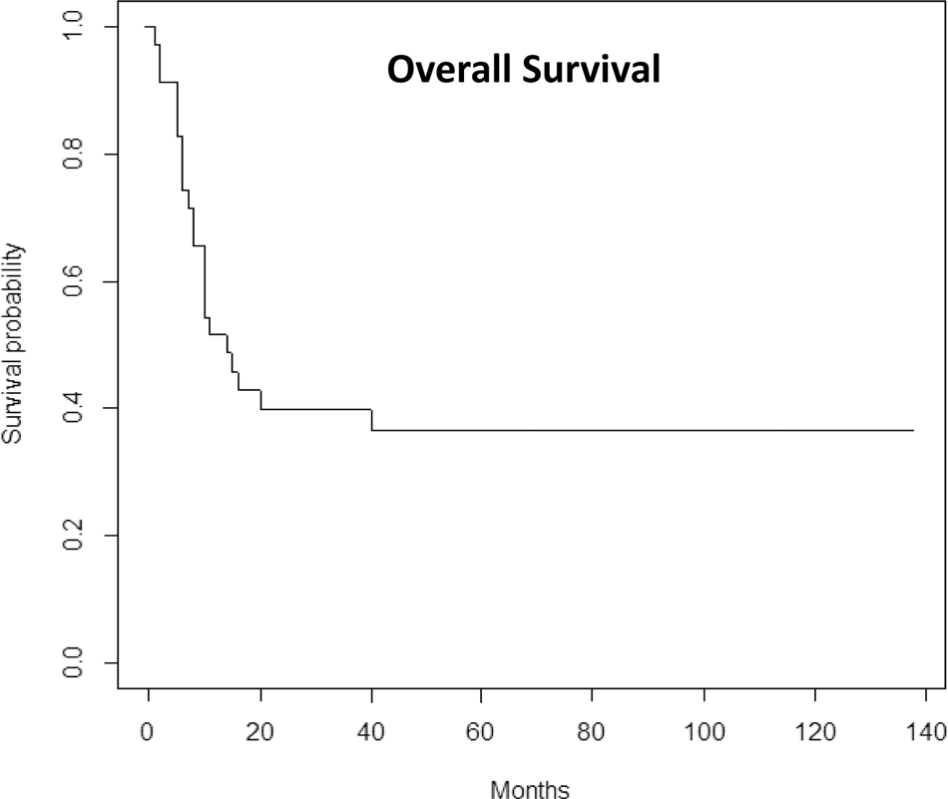
Overall survival of MLFS following HSCT. Survival curve as estimated by the Kaplan-Meier product-limit method for all 35 patients undergoing allogeneic HSCT while in MLFS.

With a median follow-up of survivors of 5 years, the median OS was 14 months (51.4% at 12 months; 39.8% at 24 months) and 13 (37%) were alive at 5 years (Fig. 1). Eight patients progressed, most within the first year. Causes of death included 29% infection^9^, 29% progression^9^, and 6%^2^ from GvHD. Notably, none of the patients in the MLFS cohort who were severely aplastic with ANC < 200 survived long term (0/8) as compared to 13/27 with MLFS following the original Cheson et al. definition. The cause of death in the aplastic subgroup was secondary to infection (5/8), progression of disease (2/8), and GVHD (1/8).

## Discussion

Clinicians are often faced with having to make the decision whether to proceed with allogeneic transplant in those with MLFS in the face of incomplete data and limited guidance in the literature^9–11^. A patient with aplastic marrow may not always have certain cytogenetic or molecular features available and thus clinical insight weighs more heavily in treatment decisions. Thus, despite the heterogenicity of our group, we describe their outcomes to help guide clinicians treating this unique population.

Recent literature has supported the concept that CR may not be necessary for allogeneic HCT^4,12,13^. Unfortunately, the heterogeneity in the classification of disease status at transplant between NCCN, CIBMTR, and standardized 2003 IWG criteria has made it challenging to evaluate specific outcomes within the MLFS population, thereby frustrating the development of evidence-based treatment guidelines. Our data support our hypothesis that MLFS following the formal Cheson et al definition is sufficient for successful allogeneic hematopoietic stem cell transplant, resulting in long-term remission in a substantial portion of patients^12–15^. Our findings notably highlight that the segment of MLFS deemed aplastic with ANC < 200 are particularly high risk and have poor outcomes following transplant. As such, caution should be used in considering moving to an allogeneic transplant while still fully aplastic without demonstrating some decrease in leukocyte recovery.

In agreement with the reports in Vu et al., our study finds that NRM is high in the MLFS population (35% in our cohort)^4^. This may in part be a result of our patient population having a high median HCT-CI of 4. An additional finding was 71% (5/7) of those with a cord donor surviving, which is consistent with Milano et al who reported superior outcomes in MRD positive patients^15^ and deserves more attention in future prospective studies. Due to the aplastic marrow of several of our MLFS patients, MRD status was not obtained, however, this would be a key addition to future studies to help provide a stronger clinical impact and comparison between our findings and prior studies as well.

Our work is additionally limited by possible selection bias by the retrospective nature of the study and the heterogeneity in treatment methods that cannot be avoided when obtaining data retrospectively. This includes variation in clinician judgment that may have impacted the decision to transition patients to allogeneic HCT, as well as the choice of conditioning regimen or donor source. In order to reduce biases with respect to reported data, we ensured a standardized approach to our chart review with inter-rater sensitivity analysis for quality assurance.

To our knowledge, our review provides the largest cohort of patients in MLFS assessed following allogeneic HCT, though this is still a limited sample. Our ability to compare populations and make definitive conclusions is hampered by not having full NGS or molecular data on all subjects as well as by the donor and transplant induction heterogeneity. Presently, available studies on patients not in CR have proposed that failure to achieve hematologic recovery may be a strong indicator of residual disease and suggests poor outcomes, however, our data directly challenges this theory. Furthermore, our observed low relapse rates and encouraging OS are promising findings. Our data questions, however, whether some extent of hematologic recovery should be seen prior to transplant, as evidenced when comparing outcomes between those who were aplastic (ANC < 200) or not prior to HCT. Continued evaluation of this population is warranted. Additionally, future data collection looking at transplant outcomes of the other NCCN response criteria outlined in Table 1 (i.e., PIF or CRi) can help place our findings into a broader perspective as clinicians make decisions on the timing of this modality.

## Supplementary information

Supplementary Table 1
