# Supplementary material for: Morphologic leukemia-free state in acute myeloid leukemia is sufficient for successful allogeneic hematopoietic stem cell transplant
Source: Blood Cancer J. 2021 May 16;11(5):92. doi: 10.1038/s41408-021-00481-9 (PMC8126559; doi:10.1038/s41408-021-00481-9)
Supplement: Supplementary file 1 — Supplementary Table 1 [file 41408_2021_481_MOESM1_ESM.docx]

Supplementary Table 1a

**Patient and Disease Characteristics** (n=35)

| **Demographics** | |
| --- | --- |
| Male/Female | 26/9 |
| Caucasian/African-American | 31 (87%) / 4 (13%) |
| Median Age (range) | 50 years (20 - 72) |
| Median BMI (kg/m2) | 28.1 |
| **HCT-CI score** | |
| 1 | 4 |
| 2 | 6 |
| 3 | 6 |
| 4-5 | 11 |
| > 6 | 8 |
| HCTCI score median (range) | 4 (1 – 9) |
| **Disease characteristics** | |
| Relapsed or failure of at least 2 induction regimens | 35/35 (100%) |
| Normal cytogenetics | 16/33 (46%) |
| High-risk genetics (per NCCN definition) | 10/35 (29%) |
| Initial NGS | Data available for n = 17 |
| *FLT3* mut | *FLT3-ITD* 4/17  *FLT3-TKD* 2/17  *FLT3* unknown 1/17 |
| *NPM1* mut | 4/17 |
| *ASXL1* mut | 2/17 |
| *IDH1/2* mut | 1/17 |
| *NRAS* mut | 2/17 |
| *RUNX1* mut | 1/17 |
| *SRSF2* mut | 1/17 |
| *TET2* mut | 2/17 |
| *CEBPA* mut | 2/17 |
| No abnormalities | 6/17 |
| Pre-transplant bone marrow cellularity | Hypocellular (<5%): 17/35  5-50% cellular: 14/35  51-100% cellular: 4/35 |
| **MLFS Status** | |
| Aplastic marrow (<5% blasts with <200 cells) | 8/35 |
| Not aplastic (<5% blasts but >200 cells) | 27/35 |
| **CIBMTR Status at Transplant** | |
| PIF | 19/35 (54%) |
| Cri | 12/35 (34%) |
| Rel 1 or 2 | 4/35 (11%) |

Supplementary Table 1b:

**Transplant characteristics (n=35)**

| **Graft** | |
| --- | --- |
| PBPC | 27/35 (77%) |
| Cord | 7/35 (20%) |
| Bone marrow | 1/35 (3%) |
| **Donor** | |
| Matched Unrelated Adult or Cord Blood | 25/35 (71%) |
| Matched Related | 10/35 (29%) |
| Related haploidentical | 7/10 |
| **Conditioning regimen** | |
| Myeloablative Bu/cy or TBI/cy | 6 |
| Reduced intensity |  |
| Bu/Flu +/- T cell depletion | 12 |
| TBI/Flu +/- T cell depletion | 11 |
| Flu/Mel or cy | 6 |
| **Average time to transplant   (days from bone marrow biopsy)** | **29** |
